# Adapting to a global pandemic: a qualitative assessment of programmatic responses to COVID-19 in the multi-country Women’s Integrated Sexual Health (WISH) programme

**DOI:** 10.1080/26410397.2023.2260174

**Published:** 2023-10-13

**Authors:** Katy Footman, Pippa Page, Victoria Boydell, Megan McLaren, Sandra Mudhune

**Affiliations:** a Consultant, Itad, Hove, UK; PhD Student, London School of Economics, London, UK.; b Consultant, Itad, Hove, UK.; c Consultant, Itad, Hove, UK; Lecturer in Global Public Health, University of Essex, Colchester, UK; d Evidence and Impact Advisor, MSI Reproductive Choices, London, UK; e Deputy Director, Evidence and Learning – WISH2ACTION, International Planned Parenthood Federation, Nairobi, Kenya

**Keywords:** pandemic, sexual and reproductive health, adaptation, lessons learned

## Abstract

The COVID-19 pandemic caused significant disruption to sexual and reproductive health and rights (SRHR) globally but there is little published evidence on the COVID-19 response of SRHR programmes, or lessons learned through their adaptations. To document the COVID-19 response of a global SRHR programme (the Women’s Integrated Sexual Health programme), in-depth interviews were conducted between April and July 2021 with 22 key informants from implementing partners in Sierra Leone, Ethiopia and central or regional offices, the UK Foreign, Commonwealth and Development Office and the third-party monitoring partner. Framework analysis methods were used. Several rapid COVID-19 adaptations were identified: the development of crisis management and communication teams; increased partnership and engagement with government; reduced contact and risk in service delivery; reformulated community mobilisation; flexible performance management and remote methods of quality assurance; and sharing of learnings alongside the development of new guidance and tools. Throughout the pandemic, the programme was able to continue high-quality service delivery, though equity goals proved more difficult to reach. Challenges included the continually changing environment, competing pressures on governments, burdensome reporting, and staff burnout. The pandemic response was facilitated by prior experience of health emergencies, strong government relationships, a supportive workforce and some pre-existing approaches, tools, and systems. This study has identified important lessons that can inform programming in future crises, including the need for immediate recognition of SRHR as essential, sustained support for staff, use of multiple mechanisms to reach marginalised groups, adequate funding for equity goals, and a better balance between the burden of reporting and accountability needs.

## Résumé

La pandémie de COVID-19 a profondément bouleversé la santé et les droits sexuels et reproductifs dans le monde, mais on dispose de peu de données publiées sur la riposte des programmes de santé et de droits sexuels et reproductifs à la COVID-19, ou sur les enseignements tirés de leurs adaptations. Pour documenter la réponse à la COVID-19 d'un programme mondial de santé et de droits sexuels et reproductifs (le programme de santé sexuelle intégrée des femmes), des entretiens approfondis ont été menés entre avril et juillet 2021 avec 22 informateurs clés provenant de partenaires d'exécution et de leurs bureaux centraux ou régionaux en Sierra Leone et en Éthiopie, du Ministère des affaires étrangères, du Commonwealth et du développement du Royaume-Uni ainsi que du partenaire extérieur chargé du suivi. Des méthodes d'analyse du cadre ont été utilisées. Plusieurs adaptations rapides à la COVID-19 ont été identifiées : la formation d'équipes de gestion de crise et de communication ; un partenariat resserré et une collaboration accrue avec les autorités ; une réduction des contacts et des risques pendant la prestation des services ; une reformulation de la mobilisation communautaire ; une gestion souple de la performance et des méthodes d'assurance qualité à distance ; et la mise en commun des enseignements parallèlement à l'élaboration de nouveaux conseils et outils. Tout au long de la pandémie, le programme a pu continuer à assurer des services de qualité, même si les objectifs en matière d'équité se sont révélés plus difficiles à atteindre. Au nombre des obstacles figuraient l'environnement en constante évolution, les pressions concurrentes sur les gouvernements, la lourdeur des exigences en matière de rapports et l'épuisement du personnel. La riposte à la pandémie a été facilitée par une expérience antérieure des urgences sanitaires, de solides relations avec les autorités, le soutien de la main-d'œuvre et quelques approches, outils et systèmes préexistants. Cette étude a identifié d'importantes leçons qui peuvent guider la programmation lors de crises futures, notamment la nécessité d'une reconnaissance immédiate de la santé et des droits sexuels et génésiques comme des services essentiels, le soutien suivi du personnel, l'utilisation de mécanismes multiples pour atteindre les groupes marginalisés, un financement approprié pour les objectifs en matière d'équité et un meilleur équilibre entre les exigences relatives aux rapports et la nécessaire redevabilité.

## Resumen

La pandemia de COVID-19 causó una interrupción significativa en salud y derechos sexuales y reproductivos (SDSR) a nivel mundial, pero no hay mucha evidencia publicada sobre la respuesta de los programas de SDSR a COVID-19, ni sobre las lecciones aprendidas por medio de sus adaptaciones. Para documentar la respuesta a COVID-19 por un programa mundial de SDSR (el programa Salud Sexual Integrada de la Mujer), se realizaron entrevistas a profundidad entre abril y julio de 2021 con 22 informantes clave de socios ejecutores en Sierra Leona, Etiopía y oficinas centrales o regionales, la Oficina de Relaciones Exteriores, Commonwealth y Desarrollo del Reino Unido y el socio externo de monitoreo. Se utilizaron métodos de análisis de marco. Se identificaron varias adaptaciones rápidas a COVID-19: la creación de equipos de gestión de crisis y comunicación; más alianzas e interacción con el gobierno, menor contacto y riesgo en la prestación de servicios; movilización comunitaria reformulada; gestión flexible del rendimiento y métodos remotos de garantía de la calidad; e intercambio de aprendizajes junto con la elaboración de nuevas guías y herramientas. Durante la pandemia, el programa logró continuar la prestación de servicios de alta calidad, aunque los objetivos de equidad resultaron ser más difíciles de alcanzar. Algunos de los retos fueron: el entorno en continuo cambio, presiones conflictivas sobre los gobiernos, onerosas obligaciones de rendición de informes y agotamiento del personal. La respuesta a la pandemia fue facilitada por experiencia anterior con emergencias de salud, sólidas relaciones con el gobierno, una fuerza laboral solidaria, y algunos enfoques, herramientas y sistemas preexistentes. Este estudio identificó importantes lecciones que pueden informar los programas en futuras crisis, tales como la necesidad de reconocer SDSR como esenciales inmediatamente, el apoyo continuo para el personal, el uso de múltiples mecanismos para llegar a grupos marginados, financiamiento adecuado para los objetivos de equidad y mejor equilibrio entre la carga de rendición de informes y las necesidades de rendición de cuentas.

## Introduction

The COVID-19 pandemic caused immediate and significant disruption to sexual and reproductive health and rights (SRHR) globally. Beyond the strain on health systems caused by COVID-19, societal responses to the pandemic, such as lockdowns and travel restrictions, forced health services to shut down or rendered them inaccessible.^1^ Although the World Health Organization (WHO) issued interim guidance for maintaining essential services during an outbreak – which included reproductive health services – there were delays or failures in many countries to define SRHR as essential, thus denying people access to time-sensitive and potentially life-saving services.^1,2^ In addition, supply chain problems, transportation issues, redirected resources and staff shortages reduced the availability and accessibility of services, while fears of infection reduced demand.^3^

Past public health emergencies illustrate the enormous indirect impacts that infectious disease outbreaks can have on SRHR, though these impacts often go unrecognised.^4^ For example, service disruptions and fear of seeking treatment in response to the 2013–2016 Ebola virus outbreak in West Africa were estimated to contribute to 3600 maternal deaths, neonatal deaths and stillbirths in Sierra Leone, which almost equals the number of deaths directly caused by Ebola in that country.^5^ Studies have observed reduced utilisation of family planning (FP) services in past outbreaks, e.g. Ebola,^6^ as well as inability of health facilities to keep up with demand for contraception.^7^

Initial conservative estimates of the potential impact of the COVID-19 pandemic on SRHR identified that a proportional decline of 10% in the use of contraceptive methods due to reduced access would result in an additional 49 million women with an unmet need for modern contraceptives and an additional 15 million unintended pregnancies over the course of a year, with disastrous implications for the lives of women and their newborns.^1^ Ministries of health and service delivery organisations reported sharp declines in access to services in the early stages of the COVID-19 pandemic, ^8–10^ with the greatest risk for vulnerable and marginalised communities, who may be less able to find or pay for alternatives.^10^ The COVID-19 pandemic therefore required organisations delivering SRHR programmes to rapidly evolve their service delivery models to respond to the crisis.

Evidence suggests that services were quickly restored, or alternatives were developed in many low- and middle-income countries, through greater use of telemedicine, home-based and community-based care and social media.^11^ However, research is needed to explore how programme adaptations have worked and the factors required for successful implementation.^11^ In other areas of health (HIV, non-communicable disease, child health), qualitative and mixed methods research has explored how programmes have adapted to COVID-19.^12–14^ Quantitative studies have also assessed the impact of COVID-19 adaptations on HIV prevention.^15,16^ However, we were only able to identify one mixed methods study which assessed adaptations to SRHR programmes beyond HIV.^17^ Similarly, while studies have assessed the impact of previous health emergencies (such as Ebola) on sexual and reproductive health (SRH) services,^5,6,18^ we did not find any studies exploring how SRH programmes adapted to these prior emergencies, and resulting lessons learned. Broader evidence is needed on the adaptations introduced in response to the COVID-19 pandemic and on the experiences and lessons learned throughout the process of adapting SRHR programmes during health emergencies.

### Aims

This study aimed to document the rapid programmatic changes made to a global SRHR programme (the Women’s Integrated Sexual Health (WISH) programme) in response to the COVID-19 pandemic. The study set out to answer the following questions. (1) Between March and October 2020, in response to the COVID-19 pandemic, how did WISH programmatic work deviate from what was originally planned? (2) What was the impact of these shifts on the overall achievement of WISH goals, and what lessons can be learned? (3) How does the WISH experience relate to wider learnings about sexual and reproductive health and rights (SRHR) programme responses and adaptations in the context of disease outbreaks?

### WISH: the Women’s Integrated Sexual Health programme

Funded by the Foreign, Commonwealth and Development Office (FCDO), the WISH programme is the United Kingdom (UK) government’s largest, and only global, programme to support integrated SRHR. The programme was implemented in 27 countries across Africa and Asia, started in September 2018 and is expected to close in March 2024. The WISH programme aimed to deliver 24.4 million couple-years of contraceptive protection (CYPs) and reach 3.6 million additional family planning users, with the intention of averting 29,000 maternal deaths, 4 million unsafe abortions, 11 million unintended pregnancies and 17.8 million additional disability-adjusted life years. The central activities and outputs of the WISH programme are:

1. Strengthening individual knowledge and choice and building community support for SRHR
2. Driving sustainability and national ownership of SRHR programmes through supportive legal, financial and policy frameworks
3. Improving access to, and expanding choice of, voluntary family planning and SRH services
4. Increasing women’s choice and access to SRHR services through evidence-based innovations and best practice.

WISH is split into two separate consortia (Table 1). In addition, WISH4Results (W4R), led by Oxford Policy Management and Itad, is a third-party monitor (TPM) responsible for independent, ongoing monitoring of WISH and for incorporating learning throughout implementation. The programme was focused specifically on serving marginalised groups (young people, people living in poverty, and people living with disabilities). WISH is a payment-by-results (PbR) programme, with payment of all fees linked to the delivery of agreed key performance indicators (KPIs).

**Table 1.** Summary of the WISH programme consortia

Lead	Consortium 1	Consortium 2
	MSI Reproductive Choices (formerly Marie Stopes International)	International Planned Parenthood Federation
Consortium partners	International Planned Parenthood Federation, Options, Leonard Cheshire, Ipas, ThinkPlace, DKT International	MSI Reproductive Choices, Options, Humanity and Inclusion, Development Media International, International Rescue Committee
Focus regions	West and Central Africa/Sahel	East/Southern Africa and Asia
Countries	Burkina Faso, Cameroon, Chad, Côte d'Ivoire, Democratic Republic of the Congo, Ghana, Mali, Mauritania, Niger, Nigeria, Senegal, Sierra Leone	Afghanistan, Bangladesh, Burundi, Ethiopia, Madagascar, Malawi, Mozambique, Pakistan, Somalia, South Sudan, Sudan, Tanzania, Uganda, Zambia, Zimbabwe

## Methods

The study used key informant interviews with WISH stakeholders to document the rapid programmatic changes made in response to the COVID-19 pandemic and the experience of implementing these programme adaptations in two focus countries and across the global programme. Key informant interviews with programme staff were used in order to gain a detailed understanding of their lived experience of implementing the programme through COVID, the decisions that informed changes to the programme design and the challenges and facilitators for implementing these adaptations. The study period was March–October 2020 as the focus was on the immediate and rapid adaptations made at the start of the pandemic.

### Study sites

Two focus countries (Sierra Leone and Ethiopia) were selected to provide an in-depth exploration of how WISH country consortia adapted their programmes. The countries were selected based on the extent to which the programmes had to be redeveloped in response to COVID-19, their involvement in existing studies, their experience with previous disease outbreaks, the severity of infection rates, and the extent of movement restrictions and lockdowns implemented in government responses to the pandemic. Table 2 contains further information about the WISH programme in each country. Interviews were also conducted with implementing partners at the global and regional levels of the WISH programme.

**Table 2.** Summary of WISH programme partners and activities in case study countries

	Ethiopia	Sierra Leone
Lead WISH partner	IPPF: The Family Guidance Association of Ethiopia (FGAE)	Marie Stopes Sierra Leone (MSSL)
Consortium partners in country	Marie Stopes International Ethiopia International Rescue Committee Humanity and Inclusion Development Media International	Leonard Cheshire Disability
Programme activities	Build capacity of public and private facilities for SRH care Deliver services through mobile outreach teams and social franchised clinics Strengthen inclusivity of services Increase awareness of SRH services through mass media, interpersonal communications and community-based mobilisers Work closely with government to identify and act on policy or legislation needs	Delivery of FP and SRH services through 11 mobile outreach teams Awareness raising about SRH through outreach, community-based mobilisers, schools engagement and social media, with a focus on traditionally underserved groups Strengthen inclusivity of services Advocacy in partnership to further progress of the Safe Motherhood Bill and inclusion of comprehensive sexuality education in the school curriculum

### Data collection

For the key informant interviews (KIIs), participants were purposively sampled and invited to participate by the study lead (PP) in consultation with co-investigators (SM and MM) from each of the lead WISH partners, based on alignment between their roles and the types of information required to fulfil the study objectives (Table 3). When initially approached, participants were fully informed about the purposes of the research, confidentiality, the expected duration of the interview, and any potential risks or benefits. Written informed consent was sought for each participant.

**Table 3.** Organisations of study key informants

Stakeholder category	Sub-category	No. participants
WISH Implementing Partners (IPs)	Ethiopia programme	5
	Sierra Leone programme	4
	Central or regional offices	7
Foreign, Commonwealth and Development Office (FCDO) staff responsible for overseeing the WISH programme		3
WISH4Results staff		3
**Total**		**22**

Data collection took place between April and July 2021. KIIs were conducted by the study lead (PP) and a research assistant using a topic guide. The topic guide included questions about the overall management of the programme during COVID-19, the implications of the pandemic for delivering the programme’s original mandate, the programmatic adaptations made by WISH partners, the toll of recalibrating and adapting WISH programmes, and the impact on staff. All interviews were conducted remotely in English and were audio recorded (with permission) using inbuilt recording functions within Microsoft Teams or Zoom. Interview recordings were transcribed by the interviewer (PP).

The study design was informed by a literature review on SRHR programme adaptations to disease outbreaks, which highlighted the lack of studies assessing how SRHR programmes have adapted to COVID-19, or prior health emergencies, as outlined in the introduction. A review of WISH programme documentation – including routine programme reports, COVID-19 guidance developed for the programme and adapted workplans – was also conducted. These programme documents were used to triangulate findings from the KIIs.

### Data analysis

Framework analysis was used to describe the findings from the interviews because it offers a systematic method and was flexible enough to allow non-interview data to be considered in the analysis.^19,20^ After gaining familiarity with the data, a thematic framework was developed, based on an initial review of a sample of transcripts and the topic guide. The framework was then applied to each transcript by charting relevant quotes and discussion points from each interview into an Excel spreadsheet according to the structure of the broader framework. A summary for each theme was then written, and some themes were collapsed or further expanded based on patterns identified within the data.

### Ethics statement

We did not seek ethical approval due to the nature of the study: interviews covered non-sensitive questions about programme interventions and did not pose more than minimal risk to human subjects, the research was considered a routine monitoring exercise of programme adaptations, and participants were all internal staff or funders of the programme. However, ethical principles and standards were adhered to in conducting the research, including obtaining informed consent from participants, maintaining privacy and confidentiality of participants, complying with data protection legislation, and identifying potential risks to participants and mitigation strategies.

## Results

The findings and their interpretations are presented across four themes: (1) adaptations to the WISH programme; (2) outcomes of COVID-19 programme adaptations; (3) challenges for programme adaptations; and (4) facilitating factors for programme adaptations. Each theme has several sub-themes, which are discussed in detail in the sections below, and listed in Table 4.

**Table 4.** Summary of themes and sub-themes

Adaptations	Outcomes	Challenges for adaptation	Facilitators for adaptation
Development of crisis management teams and communication Partnership and engagement with government Continued service delivery with reduced contact and risk Reformulated community mobilisation to include messaging on the pandemic, using door-to-door visits, social media and radio Flexible performance management and remote methods of quality assessment, supervision and validation Sharing learnings and development of new guidance and tools	Service delivery continued and CYP goals were met Low rates of COVID transmission Innovative new ways of working developed Speed of adaptation was rapid High number of advocacy gains still achieved Equity goals (poverty, youth) were more difficult to reach Some challenges with online quality assurance/data verification Pandemic preparedness Self-care progress was limited	Continuous changing environment made planning difficult Pressure on government made it difficult to collaborate Community hostility to health workers Procurement of infection prevention supplies Burdensome reporting despite flexible performance management Staff burnout	Prior experience with Ebola and other humanitarian response situations Pre-existing relationships with government Existing community mobilisation approaches Pre-existence of certain tools and guidelines Strong data systems Supportive and committed workforce Flexible management Consortia approach of WISH (broad technical expertise)

### Theme 1: adaptations to the WISH programme

Key informants described the immediate impact of COVID-19 on the WISH programme and their broader organisations. These impacts included clinic closures, supply chain disruptions, reduced demand for services due to fear of infection, concerns that service delivery could increase COVID-19 transmission, inability to travel for service delivery or for quality assurance (QA) activities, inability to continue community mobilisation due to restrictions on gatherings and school shutdowns, and demand for health workers to join the COVID-19 response. To respond to these challenges and ensure continued service delivery, participants described the key adaptations that were made to the WISH programme, and these sub-themes are summarised below.

#### Crisis management and communication

Across the consortium, COVID-19 coordination teams, taskforces or crisis management teams were rapidly established across different partners, offices or departments. These groups would closely monitor the changing COVID situation, assess risk, make decisions on adaptations and, in some cases, release guidance. For example, in Sierra Leone a crisis management team, chaired by the country director, met twice a week and was responsible for reviewing the latest local information on COVID-19, assessing risk, formulating strategies and drafting measures. One participant in Sierra Leone described how these decisions were informed by discussion with frontline workers and that most adaptations were shared during meetings to facilitate consultation with wider team members.

#### Increased partnership and government engagement

Though advocacy was already a key component of WISH, it became even more important to engage with governments during the pandemic, so that SRHR services were recognised as essential and programmes could continue to deliver services despite movement restrictions. Government engagement was also important to further the potential of self-care (e.g. to obtain approvals for the contraceptive injection Sayana Press, or telemedicine). Country teams ensured strong relationships with the government by supporting the development of COVID-related national guidelines and policy documents and by supporting government efforts to provide information about COVID-19 to the public:

*“In some cases we were moving with the government, supporting with vehicles or messaging – using contact centres to help give out info. That really positioned us as allies to the government and then enabling [sic] us to continue operating.”* (WISH implementing partner, Global)

Programme staff coordinated closely with national efforts by taking part in national taskforces or technical working groups. Country teams also integrated elements of their programme with local public health services in some countries; for example, Marie Stopes Sierra Leone worked closely with district nurses in public health units to agree which local areas the outreach team could visit – avoiding COVID-19 “hot spots” – to provide local health talks and sensitisation and to ensure aligned messaging with youth leaders, community mobilisers and local chiefs.

#### Reducing contact and risk in service delivery

To continue service delivery while minimising risk of transmission, services were restructured to use triage, outdoor spaces and physical distancing. Refresher trainings were held for staff on infection prevention protocols. For static centres, in some cases, facility staffing levels were reduced to facilitate physical distancing, while for mobile teams there was a focus on adapting travel plans to avoid visiting communities with COVID-19 outbreaks. Services included clear messaging on why protective equipment was worn, to ensure communities felt comfortable and reduce anxiety. Infection prevention guidelines were also disseminated in communities and face masks/handwashing facilities were provided to clients. There were efforts to increase the programme focus on self-care through telemedicine and pharmacy services, with telemedicine pilots in Pakistan, Sudan and South Sudan. There were also adaptations in terms of which family planning methods could be offered, with increased access to longer-acting contraception – due to concerns about possible clinic closures – and tubal ligation services being halted in order to reduce transmission risk.

#### Reformulated community mobilisation

Community mobilisation was rapidly adapted, as restrictions on mass gatherings meant that traditional methods of in-person mobilisation and group education sessions were no longer feasible. Informants described a shift to physically distanced door-to-door work using community mobilisers, promotion of contact centres and hotlines, and increased reliance on social media and radio spots:

*“All the community mobilisation and demand generation that the MAs [Member Associations] usually do was suddenly not possible anymore […] Some of them went full-on on social media. Many signed partnerships with local radio to broadcast messages in their local language. Many also went door to door, one-on-one and small gatherings, and trying to work even more with community mobilisers.”* (WISH implementing partner, Global)

The content of mobilisation efforts also had to be adapted to introduce new messages about COVID-19 and to reassure potential users that services were still open and that extra precautions were in place to keep clients safe. For example, in Ethiopia, radio campaigns planned by Development Media International were edited to include COVID-19 messaging, and radio broadcast calendars were amended to remove messaging that was no longer relevant due to government restrictions (e.g. radio spots that were based in a school context were dropped due to school closures). Consortium partners with a technical focus on disability supported the development of inclusive communication materials and practices e.g. disability-inclusive radio spots, adapted face mask use for those who need to lip read, communications in Braille.

#### Flexible performance management and remote methods of supervision

Performance measurement and management were also adapted, as the WISH programme implementing partners are reimbursed on a payment by results basis. FCDO recognised early on that this exposed implementing partners to high levels of financial risk and insecurity in a pandemic context. A six-month waiver on key performance indicators was applied, and new rigorous reporting mechanisms were put in place instead: COVID-19 adaptation indicators were added to the log frame for quarterly reporting, and a biweekly meeting and associated RAG (Red-Amber-Green) matrix was used to regularly assess adaptations and key risks.

In programmes, there was a shift to remote supervision and management systems. Restrictions on in-person meetings and travel meant that many performance management activities needed to take place remotely for the first time, including quality monitoring, financial audits, data verification and supportive supervision:

*“Our Program Officers are supposed to provide supportive supervision and coaching and mentorship. This has to continue in order to maintain quality of service. There was a complete shutdown for a couple of weeks, so travel from Addis to the other side of the country was forbidden. We developed a remote tool – our Program Officer would sit in their home, and then called providers and provided remote support.”* (WISH implementing partner, Ethiopia)

Other examples of adaptations to quality-of-care assessments included sharing of photos or videos of service delivery or phone interviews with providers, while verification activities were adapted so that scans of randomly selected paper documents were requested and were then compared to digital reports. There were also efforts to increase remote communication to support team members through group calls, WhatsApp groups and welfare calls.

#### Sharing learnings and development of new guidance and tools

Various new forms of guidance were developed by the WISH partners to support technical functions in the context of the pandemic. Examples included guidance on procurement of essential supplies, opportunities in digital health, COVID-19 infection prevention standards for mobile outreach, COVID-19 protection for community mobilisers, inclusive remote communication, and community engagement through each phase of a pandemic. Existing guidelines (e.g. WHO, national guidelines) were also used to support the development of safe service delivery through the pandemic. Informants also discussed how information about COVID-19 and associated programme adaptations were rapidly shared between teams and programmes. For example, national programme staff put measures in place to constantly monitor COVID-19 infection rates by community or region to inform service delivery plans. Regular meetings between consortium partners offered opportunities for new practices and learnings to be shared informally between national programmes.

### Theme 2: outcomes of COVID-19 programme adaptations

Key informants described both positive and negative outcomes from the programme adaptations brought about by the COVID-19 pandemic. Sub-themes included: continuation of service delivery; difficulties reaching equity goals; low rates of COVID-19 transmission; innovative new ways of working developed; challenges with online quality assurance and data verification; rapid speed of adaptation; pandemic preparedness; advocacy gains; and limited progress towards self-care. These sub-themes are summarised in more detail below.

Programme adaptations made by WISH partners to respond to COVID-19 were described by participants as being successful in many ways, but most importantly in terms of reaching programme goals for numbers of services delivered. Although there were sudden declines in the number of clients served by the programme in the second quarter of 2020, participants described a strong recovery in the third quarter, with metrics such as CYPs reverting to normal levels.

However, while overall WISH goals to reach young people and people living in poverty were ultimately achieved, some participants perceived that the pandemic had a more significant impact on equity goals in some countries:

*“On CYPs [couple years of protection] we ramped up very rapidly, but for the other KPIs [key performance indicators] it’s been a bit more difficult. I’m thinking specifically about youth and poverty. Doing CYPs is quite easy but reaching the youth when schools and universities are closed is very difficult. Same with reaching the poorest when you can’t travel and go to places where people live.”* (WISH implementing partner, Global)

In addition to travel restrictions, increased financial strain on households due to the pandemic was seen as another challenge to reaching people living in poverty. In Ethiopia, one participant explained how the reliance on schools to engage with young people, along with a lack of resources to change strategy, impacted access to services:

*“When the project was designed, the programme depended on schooling to engage youth. That really impacted and challenged our performance in general. During the COVID-19 adaptations, we tried as much as possible to reach them using the same strategies. It didn’t work, we had to change our strategy but resources were too limited. That was a turning point on the negative side for our strategy in relation to youth.”* (WISH implementing partner, Ethiopia)

However, the pandemic also created opportunities to reach different populations, as one participant reflected that the shift from community meetings to household visits in Nigeria meant that married adolescents were more likely to access services.

Several participants also discussed the success of the WISH adaptations in terms of prevention of COVID transmission among staff and clients, despite the continuation of service delivery. Participants from the two main implementing organisations described a very low rate of COVID infection among staff, and no record of any infection of clients through service provision, as well as increased adherence to guidance and protocols on infection prevention and hygiene.

Participants also held up innovation itself as a positive outcome of the COVID-19 response, as programmes were forced to develop, sometimes in ways that could be useful regardless of pandemic conditions: “*These changes can still dominate how we work in years to come – it was a really good opportunity by country programmes to rethink how we operate*” (WISH implementing partner, Global). An example of this is the shift into remote quality assurance systems and use of remote training:

*“We tried to speed up implementing activities by doing them online. It was a new thing we experienced after the COVID-19 period. We really only thought we could implement physically beforehand in terms of monitoring, training [QA]. Now [we are] doing them virtually.”* (WISH implementing partner, Sierra Leone)

Broadly there was recognition that the shift to remote working had several benefits for programmes, including increased participation and reach and inclusion for trainings and learning events. However, participants noted some downsides of remote working, around relationship building. The third-party monitor participants also noted pushback on some methods (e.g. provider interviews) used to remotely assess the quality of care – as findings were not based on in-person observation – and also discussed the difficulties of remote data verification, as the element of surprise is lost. Additionally, while remote working reduced travel costs, remote methods of quality assurance and data verification were much more time-consuming, so that overall costs and staff time were not necessarily reduced.

The speed with which the programmes adapted was mentioned by some participants as a success of the WISH COVID response, and the programme’s “*ability to work quickly with partners and in FCDO, and with downstream partners, to have a quick and nimble response in place*” (FCDO).

However, some participants acknowledged that their organisations could have been better prepared for a pandemic which took hold globally as rapidly as COVID-19.

Advocacy gains were also mentioned by partners as an indicator of the successes of WISH adaptations. These gains included a national decree on SRHR and specific budget line item for family planning in Chad, and ministerial approval for a law on gender-based violence in Mauritania.

However, plans to expand self-care and telemedicine had to be scaled back due to the regulatory burden involved:

*“They realised there’s a whole ecosystem to do this [self-care] in terms of regulation and task shifting and training. Where possible, they tried to use self-care products, e.g. in pharmacies and used call centres a lot, but it was not as ambitious as originally planned. Because of all of those ecosystem constraints.”* (KII 64, FCDO)

### Theme 3: challenges for programmatic adaptations

Key informants described several challenges for the adaptations introduced in response to the pandemic. Sub-themes included: continually changing environment; pressure on government; community hostility; procurement of infection prevention supplies; burdensome reporting and staff burnout. These sub-themes are described in more detail below.

First, the evolving situation required constant adaptation, for example with changes in messages as the caseload shifted or when there was a spread of new misinformation. Activities were regularly being introduced and then scaled back again, which posed challenges for monitoring and auditing and made it difficult to plan expenditure and budget for new activities.

Although responding to COVID-19 required high levels of engagement with government, this was challenging given the pandemic pressures and new fiscal constraints that governments faced. In some countries the pandemic was used as “*a means to roll back on citizens’ rights on [a] whole range of areas, including women’s [rights]; and programmes changed goals and targets therefore to focus on protecting basic rights on SRHR*” (FCDO) rather than furthering these rights. Government engagement posed particular challenges for the self-care agenda, as this required more regulatory change than anticipated.

A specific challenge identified by key informants working in Sierra Leone was community hostility to health workers. There was a belief among individuals in some communities that health workers were injecting people with COVID-19, and this misperception needed to be carefully managed through wider community engagement and partnerships: “*When COVID-19 broke out, some communities thought […] they were coming with viruses and injections. They became hostile*” (WISH implementing partner, Sierra Leone).

Participants acknowledged that the introduction of a waiver on key performance indicators reduced the short-term level of financial risk faced by programmes. However, additional reporting processes brought in by FCDO to monitor adaptations and risk were time-consuming and created pressure on partners’ programmes to continuously adapt and report:

*“While trying to get things done, you’re constantly asked for examples and responses all this time, and we had so many meetings […] I understand where FCDO were coming from. They needed to know we were still achieving with the money they were paying us. But it sometimes got to the point of being – enough now, it’s too much.”* (WISH implementing partner, Regional office)

FCDO’s approach to monitoring was informed by audits of responses to previous crises, which had made recommendations for stronger monitoring of expenditure and performance.^21,22^ One FCDO participant highlighted how useful RAG calls, routine conversations and sharing were, and would have liked additional monitoring in place. However, another key informant from FCDO felt a better reporting balance was needed on contractual necessity versus “*nice to have*” and highlighted the need for better feedback mechanisms:

*“We’re a demanding donor, but we have implementation experience (many of us) too – there’s no need to do hard sell constantly. Easier said than done, we hold the power dynamics, but we need to have a transparent conversation when FCDO are being too demanding, and partners saying ‘it’s too much’. We don’t need to conform to those stereotypes.”* (FCDO)

FCDO responded to concerns about reporting burden by reducing the frequency of RAG calls.

Finally, a challenge raised by most participants was the impact of the pandemic response on staff. Team members across each of the organisations faced a significant increase in workload amidst enormous emotional and mental strain caused by the pandemic, additional pressure in the personal lives of staff members, job instability, fear of infection, staff sickness and some staff deaths. Workloads increased for various reasons across frontline roles and office-based roles, but there was no additional funding or staffing to support the increased workload. The need for improved staff and provider support was identified as a key learning from the COVID-19 response: “*I would have done a few things differently – probably given more support in terms of supporting providers, that would have been helpful. The ideas didn’t come as strong and naturally as the ideas for operational changes*” (WISH implementing partner, Global).

### Theme 4: facilitating factors for programme adaptations

Key informants also described factors that facilitated the WISH partners’ programme adaptations through the pandemic: prior experience; relationships with government; existing approaches, tools, guidelines and data systems; supportive and committed workforce; flexible management; and the consortia approach.

Although one key informant noted that no one had the experience of working in a global pandemic, many team members and partners had previous experience of public health and humanitarian emergencies. Participants identified that this helped them recognise the need to act quickly and understand the risks of the pandemic for SRHR:

*“IRC [International Rescue Committee] was a key resource for us. They had responded to Ebola and other situations. They were experienced in this quick response and flagging different levels of risk programmatically […] there was already guidance and preparedness on infection prevention and keeping staff and clients safe.”* (WISH implementing partner, Regional Office)

In Sierra Leone participants had experience of delivering services through the Ebola epidemic, which informed their COVID-19 response. For example, programme implementers collaborated closely with public health facilities, supported strong community engagement through local community health workers, worked with village leaders to build trust and address misinformation, prioritised staff safety, ensured adequate stocks of infection prevention supplies and were able to quickly adapt infection prevention protocols. This was particularly important for addressing the issue of community hostility.

Although there were high levels of strain on governments, which made collaboration difficult, strong pre-existing relationships with the government facilitated COVID-19 responses in some countries. Many countries were quick to acknowledge SRH as essential services, and there was also some progress towards self-care in some countries. For example, in Madagascar self-treatment guidelines were approved, as “*those discussions had already started and it was ongoing […] They’re not going to engage in something you think is great but they’ve not heard before*” (WISH Implementing partner, Regional office).

Some participants described how existing community mobilisation approaches offered a platform for adaptation through the pandemic, such as a youth network in Sierra Leone and existing IRC-coordinated community outreach activities in refugee camps in Ethiopia. The existence of certain tools and guidelines was also reported to facilitate the programmatic adaptations, such as existing clinical guidelines on infection prevention, as well as strong data systems which did not need to be amended dramatically to reflect changes in programmes.

Some participants also recognised that the supportive and committed workforce was a key facilitating factor: “*Countries just had an attitude of getting on and doing it, we’re not going to just stop*” (WISH implementing partner, Regional). One participant reflected that team members were concerned about COVID-19 infection risk and the potential impact on livelihoods, which meant that there was high support for measures to reduce the spread of COVID while continuing service delivery.

Some participants also described attributes of the WISH programme management and structure that facilitated the rapid response to the pandemic. The KPI waiver was mentioned by participants from both FCDO and the WISH programme, as the waiver increased confidence and autonomy for programmes, though only in the short term.

The consortia approach of WISH and the broad technical expertise also facilitated COVID-19 adaptations as partners had different specialities, such as humanitarian response, disability and inclusion.

## Discussion

At the onset of the COVID-19 pandemic, health programmes in low- and middle-income countries needed to respond rapidly to a drastically altered global and local landscape. This paper outlined key adaptations introduced by the UK Government-funded “Women’s Integrated Sexual Health” programme to ensure continued access to SRH care through the crisis. The findings highlight how SRH programmes can continue, strengthen and innovate through public health emergencies. Similarly, previous research on the impact of Ebola on contraceptive use in West Africa indicated that the FP sector can recover and even improve following such significant disruption.^18^

The COVID-19 adaptations and facilitators identified in our study also reflect some of those reported in recent publications and studies, including the importance of community-level services,^15,23^ positive impacts of online mentoring,^24^ improvements in infection prevention,^23,25^ importance of coordination between partners and government^23^ and increased use of digital health interventions.^25^ Challenges identified in our study have also been reported elsewhere, such as misinformation about COVID-19,^26^ difficulties with the shift to online modes of working,^25^ a lack of external funding and support^17^ and the need for improved support for the health workforce, including physical protection, mental health needs and capacity to assume new tasks.^23,25^

Although not seen in our study, research from a youth service in Zimbabwe found that COVID-19 infection control measures restricted the effectiveness of and engagement with the intervention, as time with healthcare providers was reduced and social activities were removed.^27^ Similar findings were reported from a review of teenage pregnancy services in Sierra Leone,^26^ suggesting the need for careful balancing of physical risk with social risk, particularly for adolescent services.

Telemedicine and digital health interventions have been a common adaptation to COVID-19 among SRH programmes, though most of these innovations have been documented by the private sector and there is limited evidence on the effectiveness and sustainability of these approaches.^11^ In our study, telemedicine and digital health interventions were seen as important innovations, but policy barriers had reduced their potential impact and there were also concerns about their ability to improve access for rural populations. A recent review of health system readiness to expand telemedicine post-pandemic in Africa similarly identified that despite high willingness to adopt telemedicine and recognition of its potential to improve health care access, there is a need for improved government support.^28^ The need for faster implementation of policy changes to facilitate continued access to SRH services during the pandemic (e.g. through telemedicine), has also been identified in previous research.^9^ Strong government relationships and engagement can enable SRH programmes to continue to deliver essential services through infectious disease outbreaks. However, there is also a clear need for regulatory bottlenecks to be removed to enable innovative and resilient models of care that can address SRH needs (e.g. approval of remote consultations, removal of unnecessary clinical tests, approval of task-sharing).^1^ During an emergency response, regulatory change can be very difficult to achieve, so it is critical to continue work to build an enabling environment for self-care as the pandemic eases.

Public health emergencies can have enormous indirect impacts on SRH,^4^ so it is vital to learn from past outbreaks.^1^ This study aimed to reflect and gather learnings from the WISH programme experience of adapting through COVID-19. From a policy perspective, one of the key learnings from the pandemic was the need for faster and greater recognition that SRH is essential health care. This study also identified operational implications for future public health emergencies and disease outbreaks. These include the need to maintain a focus on equity during emergency response, which may require additional funding during periods of crisis. Programmes may increase their resilience in this area by having multiple mechanisms for reaching marginalised groups, in order to avoid reliance on only one approach (e.g. schools for reaching adolescents). Echoing previous reports,^24^ the study also underlined the importance of stronger and sustained support for staff, particularly through periods of immense disruption due to the high levels of pressure and burnout experienced. WISH also highlighted operational learnings for non-pandemic times, including the potential for remote learning, supervision and quality assurance to improve engagement and inclusivity. However, these methods are not without challenges, and the workload and costs involved in verification and quality assurance are not necessarily reduced when using remote methods, which needs to be considered for future programmes. Finally, the study identified key learnings for donors around programme management, as the need for accountability needs to be balanced with the burden on implementing partners, particularly during emergency situations or periods of unrest.

### Limitations

The study had some limitations. First, due to the scale of the WISH programme, it was not possible to capture and report the detail of all the adaptations introduced throughout the COVID-19 pandemic, so this study represents a broad overview of the WISH programme experience through COVID-19. Second, due to feasibility constraints the interviews focused on two countries, which may not be generalisable to other settings where WISH was operating, though participants with global or regional roles did provide a wider perspective. Third, the study period focused on March–October 2020, and – given the dynamic and shifting nature of the response to the COVID-19 pandemic – the study’s findings may not reflect the impact of later phases of the pandemic on SRHR programming. The authors of this paper were employed or contracted by organisations under the WISH programme through UK FCDO, which may have introduced bias into the design and implementation of the study. Further, as participants were all closely involved in the management and implementation of the WISH programme, there may have been some social desirability bias in their responses or some avoidance of discussing organisational sensitivities – though all participants were assured that their responses would be anonymised. We were not able to interview service users or other stakeholders due to time and budgetary constraints, but our goal was to explore the experience of adapting to the COVID-19 pandemic from an implementation perspective, rather than to evaluate the impact of these adaptations on service users.

## Conclusion

The COVID-19 pandemic caused significant disruption to SRH programmes in low- and middle-income countries. Programmes required continuous and sometimes drastic adaptation. Through strong engagement with government, reduced transmission risk in service delivery, and adaptations to community mobilisation and performance management, the WISH programme was able to continue to deliver high-quality SRH services. However, it was more challenging to continue to reach the programme’s equity goals, which may reflect the need for additional resources and a broader range of mechanisms to safeguard access for marginalised groups during periods of crisis. Many challenges were identified, including staff burnout, burdensome reporting, community hostility to health workers, and difficulties collaborating with government due to pandemic pressures. Yet the programme’s fast, innovative and rigorous response to the pandemic was facilitated by prior experience with public health emergencies and humanitarian response; strong pre-existing relationships with government, which enabled the recognition of SRH as essential services; the consortium approach; a supportive workforce; and some pre-existing approaches, tools and systems. Protection of SRHR and continued access to SRH services are essential during public health emergencies and crises. This article provides important lessons learned from the COVID-19 response, which can inform programme operations through future crises.
